# Urban-Rural Disparity of Generics Prescription in Taiwan: The Example of Dihydropyridine Derivatives

**DOI:** 10.1155/2014/905213

**Published:** 2014-02-10

**Authors:** Chia-Chen Hsu, Chia-Lin Chou, Shu-Chiung Chiang, Tzeng-Ji Chen, Li-Fang Chou, Yueh-Ching Chou

**Affiliations:** ^1^Department of Pharmacy, Taipei Veterans General Hospital, Taipei 11217, Taiwan; ^2^School of Medicine, Institute of Hospital and Health Care Administration, National Yang-Ming University, Taipei 11221, Taiwan; ^3^Department of Family Medicine, Taipei Veterans General Hospital, Taipei 11217, Taiwan; ^4^Department of Public Finance, National Chengchi University, Taipei 11605, Taiwan; ^5^Department and Institute of Pharmacology, National Yang-Ming University, Taipei 11221, Taiwan; ^6^College of Pharmacy, Taipei Medical University, Taipei 11031, Taiwan

## Abstract

The aim of the current study was to investigate the urban-rural disparity of prescribing generics, which were usually cheaper than branded drugs, within the universal health insurance system in Taiwan. Data sources were the cohort datasets of National Health Insurance Research Database with claims data in 2010. The generic prescribing ratios of dihydropyridine (DHP) derivatives (the proportion of DHP prescribed as generics to all prescribed DHP) of medical facilities were examined against the urbanization levels of the clinic location. Among the total 21,606,914 defined daily doses of DHP, 35.7% belonged to generics. The aggregate generic prescribing ratio rose from 6.7% at academic medical centers to 15.3% at regional hospitals, 29.4% at community hospital, and 66.1% at physician clinics. Among physician clinics, the generic prescribing ratio in urban areas was 63.9 ± 41.0% (mean ± standard deviation), lower than that in suburban (69.6 ± 38.7%) and in rural (74.1% ± 35.3%). After adjusting the related factors in the linear regression model, generic prescribing ratios of suburban and rural clinics were significantly higher than those of urban clinics (*β* = 0.043 and 0.077; *P* = 0.024 and 0.008, resp.). The generic prescribing ratio of the most popular antihypertensive agents at a clinic was reversely associated with the urbanization level.

## 1. Introduction

Owing to geographic and socioeconomic factors, rural residents have usually less satisfactory access to various kinds of health care services than urban counterparts. In the past, many investigations into urban-rural disparity had been devoted to aspects of hospitalizations [1, 2], surgical procedures [3], dental care [4], specialist care [5], emergency services [6, 7], preventive services [8, 9], nursing home care [10, 11], terminal care [12–15], complementary and alternative medicine use [16], and treatment outcomes [17]. The majority of these studies were related to undersupply of not easily transportable facilities and specialists in rural areas. In contrast, the urban-rural disparity in pharmaceutical care received less attention. A recent study about Medicare Part D in the USA had identified the role of branded drugs in regional variations of expenditure for prescription drugs [18]. However, extensive, detailed analyses of urban-rural disparity of generics prescription have not been well documented.

The aim of the current study was to investigate the urban-rural disparity of generics prescription within the universal health insurance system in Taiwan. The most popular antihypertensive drugs in the ambulatory sector would be analyzed as an example. Specifically for physician clinics, we would adjust patient, physician, and clinic features to measure the relationship between generic prescribing ratio and urbanization level of clinics.

## 2. Materials and Methods

### 2.1. Ethics Statement

This study was approved by the institutional review board of Taipei Veterans General Hospital. Since the study was a retrospective medication record review and all data were deidentified, the review board approved that written consent from patients was not required.

### 2.2. Data Sources

The single-payer National Health Insurance (NHI) system in Taiwan started in 1995 and provides comprehensive healthcare coverage for more than 99% of inhabitants (23,074,487 beneficiaries at the end of 2010, equaling 99.6% of all population) [19]. Since 1999, the Bureau of NHI has released the claims data to the public for research purpose under the project of National Health Insurance Research Database (NHIRD) managed by the National Health Research Institutes [20]. To make a balance between privacy protection and longitudinal follow-ups, the NHIRD encrypted the identification numbers of persons and healthcare facilities in the datasets consistently for each applicant, so that record linking within datasets is feasible [21].

In the current study, we obtained one special subset of NHIRD, the Longitudinal Health Insurance Database for 2010 (LHID2010), that contained claims data belonging to one million people randomly sampled from all NHI beneficiaries in 2010. The claims data were traced back to 1996 and will be followed up afterwards. According to NHIRD, there was no significant difference in the age, gender distribution, and utilization between people in the LHID2010 and those in the complete NHIRD datasets. Our analysis was limited to the year 2010 with 15,431,528 records of ambulatory visits plus 89,492,613 prescribed items and 2,920,513 records at independent pharmacies plus 10,330,875 dispensed items. Not all prescribed drug items were dispensed at independent pharmacies in Taiwan because the outpatient departments at hospitals owned pharmacies and physician clinics were allowed to have dispensing practice if pharmacists were hired [22].

To identify the accreditation status and location of medical facilities, we used the registry for contracted medical facilities. The accreditation was divided into academic medical center, metropolitan hospital, local community hospital, and physician clinic. The location could be used to identify the governing branch of Bureau of NHI. The web site of the Bureau of NHI (http://www.nhi.gov.tw/) also provides the details of each reimbursable drug item, including the brand or generic status and the code of the Anatomical Therapeutic Chemical (ATC) classification system. The defined daily dose (DDD) of each ATC code was looked up on the web site of the WHO Collaborating Centre for Drug Statistics Methodology, Oslo, Norway (http://www.whocc.no/). The 2012 version was used for DDD calculation.

Additionally for calculating the covariates, we used the registry for medical personnel to have each prescribing physician's age, the monthly claim summary for ambulatory care claims to know the monthly service volume of each clinic, the registration files of beneficiaries to identify each patient's income, and the registry for catastrophic illness patients to learn whether a patient had a heavier illness.

### 2.3. Study Design

The drug group of dihydropyridine (DHP) derivatives (selective calcium channel blockers with mainly vascular effects) was the main focus of our study. According to the drug statistics from the Bureau of NHI, one of dihydropyridine derivatives, amlodipine, has remained the most popular antihypertensive agent and ranked as the first in pharmaceutical expenses with the NHI since 2000. From the drug master files of the Bureau of NHI, 203 drug items under the ATC coding of C08CA (dihydropyridine derivatives), C08GA01 (nifedipine and diuretics), and C10BX03 (atorvastatin and amlodipine) were identified for further analysis. Not all items were available at the market in 2010.

Within the NHI, physicians could write refill prescriptions entitling the patients to obtain one month's drug supply for three consecutive months. Because patients might not redeem every refill prescription, we extracted only the dispensed items of DHP, either at hospitals/clinics or at independent pharmacies, and the related prescribing details from the LHID2010 in 2010. Thus, prescribing in our study was represented with dispensed data. For each item, we identified its brand or generic status and calculated its dispensed amount in number of DDD. Besides, we also traced the prescribing physician and the medical facility. The identification number of the medical facility was then linked to the town where the facility was located. The urbanization level of a town was determined on the basis of a study conducted at the National Health Research Institutes in Taiwan and operationally categorized into urban, suburban, or rural [23].

In the current study, we firstly computed the aggregate amounts of all and generic DHP prescriptions and stratified the data by DHP ingredient to obtain an overview of the market for DHP within the NHI. The unit at calculating drug amounts was DDD. The aggregate data were further stratified by accreditation status and urbanization level of medical facilities. Specifically for all physician clinics, we examined the hypothesis that the generic prescribing ratio (the proportion of DHP prescribed as generics to all prescribed DHP) of a clinic was reversely associated with the urbanization level. The dependent variable was the generic prescribing ratio of a clinic. The independent variable was the urbanization level of a clinic. To take possible confounders into consideration, we also included as covariates the following factors: the prescribing physician's age, the governing branch of Bureau of NHI, the average monthly number of visits of a clinic, the average monthly amount of claims, the proportions of male patients, patients exempt from copayment and patients with a heavier illness certificate to all patients receiving DHP in a clinic, and the mean age and income of patients receiving DHP. If two or more physicians worked at the same clinic, we chose the physician in charge to calculate the prescribing physician's age of a clinic under the assumption that she/he had the deciding role in compiling the formulary in the clinic. The Bureau of NHI has six branches with separate budgets. The branches are divided according to geographic areas. The medical facilities governed by the same branch share the annual budget of healthcare expenses with a floating point value (monetary conversion factor) of reimbursement. Therefore, the governing branch of Bureau of NHI was listed into covariates.

### 2.4. Statistical Analysis

Data extraction and computation were performed with the Perl programming language (version 5.14.2). SPSS software (version 17) was used for statistical analysis. Pearson's *χ* ^2^ test and analysis of variance (ANOVA) were used for group comparisons, respectively. Furthermore, we used linear regression models to compute all related measures and then adjust the other factors to test the relationship between generic prescribing ratio and urbanization level more accurately. A *P* value < 0.05 (two tailed) was considered as statistically significant.

## 3. Results

From the one-million person cohort datasets in 2010, we extracted 603,664 records of ambulatory visits with 614,334 prescribed items of DHP and 196,718 records at independent pharmacies with 198,392 dispensed items of DHP. After eliminating the unredeemed prescriptions, mainly as refill prescriptions, we obtained 750,087 prescribed/dispensed items for further analysis. These items had been prescribed to 88,900 patients by 19,156 physicians at 6,021 medical facilities. There were 109 distinct DHP items belonging to 12 ATC codes. Totally, 24 branded items accounted for 472,557 (63.0%) prescribed items and 85 generics only for 277,530 (37.0%). When the amount of prescriptions was expressed in DDD, 21,606,914 DDDs of DHP had been prescribed and generics accounted for 35.7% (Table 1). Amlodipine had the largest share (62.4%) of the total DHP amount, followed by nifedipine (13.9%) and felodipine (12.7%).

When stratified by urbanization level of medical facilities, 67.4% (14,555,983/21,606,914) of the total DHP amount had been prescribed in urban areas, 25.8% in suburban, and 6.9% in rural (Table 2). When stratified by accreditation status of medical facilities, physician clinics accounted for 40.3% of the total DHP amount, followed by metropolitan hospitals (24.1%), academic medical centers (22.5%), and local community hospital (13.1%). The aggregate generic prescribing ratio rose from 28.0% in urban areas to 66.5% in rural areas and from 6.7% at academic medical centers to 66.1% at physician clinics. Among physician clinics, the aggregate generic prescribing ratio rose from 61.0% in urban areas to 74.8% in rural areas.

To further examine the relationship between generic prescribing ratio and urbanization level in physician clinics, we calculated the individual generic prescribing ratio and other potential confounding factors of each physician clinic. The generic prescribing ratio of physician clinics in urban areas was 63.9 ± 41.0% (mean ± standard deviation), lower than that in suburban (69.6 ± 38.7%) and in rural (74.1% ± 35.3%). The differences were statistically significant (Table 3). These three groups of physician clinics also differed in monthly visit count, monthly claims amount, the governing branch of Bureau of NHI, share of patients exempt from copayment, and average patients' age. In the regression model without adjustment, generic prescribing ratios of suburban and rural clinics were significantly higher than those of urban clinics (*β* = 0.086 and 0.150 resp.) (Table 4). After adjusting the related factors which had no problematic collinearity, the strengths of relationship between generic prescribing ratio and urbanization level became smaller (*β* = 0.043 and 0.077) but were still statistically significant (*P* = 0.024 and 0.008).

## 4. Discussion

In recent years, most developed countries had faced the challenge of rising healthcare expenditure, of which drug costs accounted for a large share. Beside aging demographic structure, the pharmaceutical innovation was usually considered as a cost factor. Therefore, one of frequently implemented measures of controlling drug costs was to encourage the use of cheaper generics [24–26]. While the majority of researches into generics switch or substitution disputed about the drug quality, our current study touched the issue of equity. Our results revealed that generics of DHP were far less frequently prescribed at outpatient departments of hospitals than at physician clinics in Taiwan. Because most hospitals, also larger ones, were located in urban areas, our further analysis on physician clinics confirmed the urban-rural disparity of generic prescribing in Taiwan.

In our study, the aggregate generic prescribing ratio of DHP was 35.7%. In a US study about the Medical Expenditure Panel Survey Household Component from 1997 to 2000, 61% of drugs at outpatient sector were dispensed as generics [24]. In another US study about paid pharmacy claims from 548 self-insured employers, the aggregate generic prescribing ratio also was high as 63.3% at the end of 2009. In contrast, the aggregate generic prescribing ratio of DHP in Taiwan was relatively low despite the availability of 85 generics in all 109 distinct items. The late expiry of patent of amlodipine that occurred in 2007 did not seem to be able to fully explain the phenomenon. As compared to 35.5% of amlodipine, the generic prescribing ratio of the older nifedipine was even lower (28.8%) (Table 1).

In the discussions about the determinants of physician use of generic or new drugs, financial incentives were frequently mentioned [27, 28]. At least for physicians working at hospitals who could only prescribe drugs according to hospital formulary, the financial incentives might originate from hospital administrators instead of physicians. In our study, the average generic prescribing ratio of DHP at physician clinics in urban areas was lower than those at suburban and rural. One of potential determinants that were not listed as confounders in data adjustment was competition, among physician clinics on one hand and between physicians and neighboring hospitals on the other hand. The factor of competition was also reflected in the comparisons of generic prescribing ratio of DHP at physician clinics under different branches of Bureau of NHI. In most densely populated regions where Taipei and Northern branches are located, generic prescribing ratios were the lowest. In contrast, the ration was the highest in most sparsely populated eastern Taiwan (Table 4).

Some other factors were unmentioned in our current study, but they also remained unknown. Because physician clinics were allowed to dispense drugs in Taiwan, physicians at dispensing clinics did face financial benefits on drug choice. However, the margin of drugs, that is, the difference between the buying cost and the reimbursement from NHI, could not be known for each drug at every clinic. This factor would be further compounded by the phenomenon of “next-door pharmacy” in Taiwan, in which a physician clinic operated the neighboring pharmacy instead of hiring a pharmacist in-house [22]. Logistics of drugs were another factor hard to decipher. Although the manufacturers were specified for each drug item in the master file, the drugs were usually merchandized through distributors, especially for practicing clinics. The assortment of drugs available at a distributor would also limit the drug choice of medical facilities. The situation was conceivably more severe in rural areas where few distributors might offer services.

## 5. Conclusions

The generic prescribing ratio of the most popular antihypertensive agents at a clinic was reversely associated with the urbanization level. The underlying causes, clinical consequences, and economic justice of urban-rural disparity in prescribing generics deserve further investigation.

## Figures and Tables

**Table 1:** Prescribing amount of DHP derivatives in one million beneficiaries within Taiwan's National Health Insurance in 2010, stratified by ingredient and branded/generic status.

ATC code	Ingredient	All DHP		Branded DHP			Generic DHP		
		number of items	DDDs	number of items	DDDs	(%)	number of items	DDDs	(%)
C08CA01	Amlodipine	33	13,491,768	2	8,706,070	(64.5)	31	4,785,698	(35.5)
C08CA02	Felodipine	20	2,737,174	3	1,098,955	(40.1)	17	1,638,219	(59.9)
C08CA03	Isradipine	1	22,802	1	22,802	(100.0)			
C08CA04	Nicardipine	8	11,064	3	6,253	(56.5)	5	4,811	(43.5)
C08CA05	Nifedipine	32	3,003,654	6	2,139,413	(71.2)	26	864,241	(28.8)
C08CA06	Nimodipine	2	1,024	2	1,024	(100.0)			
C08CA07	Nisoldipine	2	3,437	2	3,437	(100.0)			
C08CA08	Nitrendipine	4	94,027				4	94,027	(100.0)
C08CA09	Lacidipine	3	510,245	1	191,513	(37.5)	2	318,732	(62.5)
C08CA12	Barnidipine	2	53,606	2	53,606	(100.0)			
C08CA13	Lercanidipine	1	1,339,212	1	1,339,212	(100.0)			
C10BX03	Atorvastatin and amlodipine	1	338,903	1	338,903	(100.0)			

Total		109	21,606,914	24	13,901,187	(64.3)	85	7,705,727	(35.7)

ATC: World Health Organization Anatomic Therapeutic and Chemical Classification; DHP: dihydropyridine; DDD: defined daily dose.

**Table 2:** Aggregate prescribing amount of DHP derivatives in one million beneficiaries within Taiwan's National Health Insurance in 2010, stratified by accreditation status and urbanization level.

	Accreditation status of medical facilities					
	Academic medical centers	Metropolitan hospitals	Local community hospitals	Physician clinics	Home care units	Total
Number of clinics						
Urban	20	54	182	2,921	5	3,182
Suburban	1	27	131	1,945	1	2,105
Rural		2	28	704		734
Total	**21**	**83**	**341**	**5,570**	**6**	**6,021**

Amount of prescribed DHP in DDD						
Urban	4,857,576	3,739,490	1,571,777	4,387,141	200	14,555,983
Suburban	3,359	1,404,477	1,055,655	3,102,206	60	5,565,696
Rural		72,611	192,764	1,219,859		1,485,235
Total	**4,860,934**	**5,216,578**	**2,820,195**	**8,709,207**	**260**	**21,606,914**

Amount of prescribed generic DHP in DDD						
Urban	324,538	646,557	428,388	2,675,767	60	4,075,110
Suburban	0	148,519	330,358	2,164,702	0	2,643,518
Rural		3,825	70,550	912,724		987,098
Total	**324,538**	**798,900**	**829,296**	**5,753,193**	**60**	**7,705,727**

Generic prescribing ratio of DHP						
Urban	6.7%	17.3%	27.3%	61.0%	30.0%	28.0%
Suburban	0.0%	10.6%	31.3%	69.8%	0.0%	47.5%
Rural		5.3%	36.6%	74.8%		66.5%
Total	**6.7%**	**15.3%**	**29.4%**	**66.1%**	**23.1%**	**35.7%**

DDD: defined daily dose; DHP: dihydropyridine.

**Table 3:** Differences in generic prescribing ratio of DHP derivatives and other measures at physician clinics of various urbanization levels.

	Urbanization level						*P*-value
	Urban (*n* = 2, 921)		Suburban (*n* = 1, 945)		Rural (*n* = 704)		
	Mean	SD	Mean	SD	Mean	SD
Generic prescribing ratio (%)	63.85	40.97	69.57	38.67	74.13	35.25	<0.001^c^
Prescribing physician's age	52.09	10.08	52.07	10.18	52.24	11.19	0.923^c^
Monthly visit count	1,668.01	1,716.78	1,693.46	1,257.96	1,450.52	932.91	0.001^c^
Monthly claims amount	836,357.00	1,330,750.00	769,430.30	976,930.10	584,243.70	492066.20	<0.001^c^
Branch of Bureau of NHI							
Taipei	1,136^a^	79.5^b^	255^a^	17.84^b^	38^a^	2.66^b^	<0.001^d^
Northern	414^a^	54.98^b^	308^a^	40.9^b^	31^a^	4.12^b^	
Middle	432^a^	35.56^b^	617^a^	50.78^b^	166^a^	13.66^b^	
Southern	329^a^	35.04^b^	383^a^	40.79^b^	227^a^	24.17^b^	
Kao-Ping	574^a^	53.2^b^	340^a^	31.51^b^	165^a^	15.29^b^	
Eastern	36^a^	23.23^b^	42^a^	27.1^b^	77^a^	49.68^b^	
Share of male patients (%)	48.22	30.13	47.15	27.56	45.75	24.65	0.091^c^
Share of patients exempt from copayment (%)	8.50	17.98	8.33	19.55	25.19	38.47	<0.001^c^
Share of patients with a heavy illness (%)	7.44	16.79	7.06	15.08	7.76	13.31	0.548^c^
Average patients' age	60.17	8.47	61.52	8.28	64.43	6.71	<0.001^c^
Average patients' income	17,359.82	15,114.28	17,505.51	11,564.02	18,166.13	7,346.10	0.345^c^

DHP: dihydropyridine; NHI: National Health Insurance; SD: standard deviation.

^a^ *n* **.**

^b^%** .**

^c^ANOVA** .**

*χ* ^2^ test.

**Table 4:** Regression model of predicting the generic prescribing ratio of dihydropyridine (DHP) derivatives of a physician clinic with urbanization level and adjusting other factors.

	Univariate			Multivariate		
	*β*	SE	*P*-value	*β*	SE	*P*-value
Urbanization level						
Urban	Reference			Reference		
Suburban	0.086	0.019	<0.001	0.043	0.019	0.024
Rural	0.150	0.027	<0.001	0.077	0.029	0.008
Prescribing physician's age	0.005	0.001	<0.001	0.004	0.001	<0.001
Monthly visit count	0.00001	0.00001	0.133	0.00005	0.00001	<0.001
Monthly claims amount	−0.0000001	0.00000001	<0.001	−0.00000009	0.00000001	<0.001
Branch of Bureau of NHI						
Taipei	Reference			Reference		
Northern	0.018	0.029	0.529	0.017	0.028	0.537
Middle	0.152	0.026	<0.001	0.160	0.026	<0.001
Southern	0.144	0.028	<0.001	0.158	0.028	<0.001
Kao-Ping	0.105	0.026	<0.001	0.118	0.026	<0.001
Eastern	0.393	0.055	<0.001	0.385	0.054	<0.001
Share of male patients	0.0002	0.0003	0.510	0.001	0.0003	0.054
Share of patients exempt from copayment	−0.001	0.0004	0.008	−0.0002	0.0004	0.650
Share of patients with a heavy illness	−0.003	0.001	<0.001	−0.001	0.001	0.015
Average patients' age	−0.003	0.001	0.005	−0.004	0.001	<0.001
Average patients' income	−0.000002	0.000001	0.002	−0.000003	0.000001	<0.001

*β*: regression coefficient; SE: standard error; NHI: National Health Insurance.
